# Community data of adults fasting for lipid and diabetes tests in Calgary, Alberta from 2010 to 2016

**DOI:** 10.1016/j.dib.2018.11.142

**Published:** 2018-12-06

**Authors:** Irene Ma, Jeannine Viczko, Christopher Naugler

**Affiliations:** a Department of Pathology and Laboratory Medicine, Cumming School of Medicine, University of Calgary, Calgary, Alberta, Canada; b Family Medicine, Cumming School of Medicine, University of Calgary, Calgary, Alberta, Canada; c Community Health Sciences, Cumming School of Medicine, University of Calgary, Calgary, Alberta, Canada

## Abstract

Data presented in this data article include the proportion and counts of community-based adult men and women who fasted for various lipid and diabetes screening-related tests from January 2010 to June 2016 in Calgary, Alberta, Canada. This data set was collected from Calgary Laboratory Service׳s Laboratory Information System, and included 3,003,667 testing encounters (1,447,720 testing encounters for men and 1,555,947 testing encounters for women) over the age of 18 years, who fasted for a lipid profile, lipid+random blood glucose (RBG), lipid+hemoglobin A_1c_ (HbA_1c_), lipid+RBG+HbA_1c_, lipid+fasting blood glucose (FBG), lipid+FBG+RBG, lipid+FBG+HbA_1c_, or lipid+FBG+RBG+HbA_1c_. Data are related to “Proportion of adults fasting for lipid testing relative to guideline changes in Alberta” (Ma et al., 2017).

**Specifications table**

**Table:** 

Subject area	*Medicine*
More specific subject area	*Laboratory Medicine; Family Medicine*
Type of data	*Tables*
How data were acquired	*Calgary Laboratory Services’ Laboratory Information System*
Data format	*Raw, analyzed*
Experimental factors	*Time since last meal for each adult was documented and defined as* ≥*8 h since last meal prior to blood collection.*
Experimental features	*Counts and proportion (mean*±*SD) of community-based men and women fasting for various lipid and diabetes screening test encounters each month in Calgary, Alberta, between January 2010 and June 2016.*
Data source location	*Calgary, Alberta, Canada*
Data accessibility	*Data are with this article*
Related research article	*This data article is submitted as a companion paper to the following research article:*
	Ma, I., J. Viczko, and C. Naugler, *Proportion of adults fasting for lipid testing relative to guideline changes in Alberta*. Clin. Biochem, 2017. 50(6): p. 344–346.

**Value of the data**

• These data of community-based adults who fasted for a lipid profile test with or without various diabetes screening tests can be used in the development of future studies in the areas of diabetes and family medicine.
• Data can be used to determine the laboratory utilization of laboratory test ordering practices by family physicians within Calgary, Alberta as well as provide as an example for other jurisdictions investigating their own laboratory utilization initiatives within Canada.
• On a national and international level, these data can assist other jurisdictions as a basis for further study as they prepare their clinics to switch from fasting-required to fasting not-required for lipid profile tests.

## 1 Data

The data presented here include the proportion (Table 1) and counts (Tables 2 and 3) of community-based women and men who fasted for a lipid profile encounter with or without various diabetes-screening related tests, which was collected from Calgary Laboratory Services (CLS) Laboratory Information System. These data were collected to document the proportion of adults fasting for a lipid profile encounter relative to the lipid guideline changes in Alberta, Canada [1].

**Table 1:** Proportion of community-based adults fasting for various lipid and diabetes screening tests over time (% mean±SD for female testing encounters; % mean±SD for male testing encounters).

**GROUP**	**2010**	**2011**	**2012**	**2013**
**Lipids**	99.39±0.330; 99.52±0.137	96.84±0.763; 97.49±0.518	95.35±0.863; 96.32±0.960	93.22±0.747; 93.88±0.670
**Lipids**+**RBG**	96.28±0.919; 96.97±0.847	88.63±1.435; 89.75±1.876	86.91±2.375; 87.98±1.982	76.72±6.748; 77.84±7.272
**Lipids**+**HbA**_**1c**_	98.86±0.521; 99.27±0.300	95.56±0.888; 96.42±0.680	93.29±1.174; 94.63±1.010	88.84±1.477; 90.83±1.470
**Lipids**+**RBG**+**HbA**_**1c**_	95.10±1.716; 94.21±3.249	91.07±2.339; 91.87±2.260	92.54±1.548; 92.22±1.006	84.67±4.006; 84.98±4.298
**Lipids**+**⁎FBG**	99.78±0.048; 99.82±0.064	98.20±0.330; 97.99±0.365	97.95±0.322; 97.71±0.329	97.76±0.394; 97.67±0.459
**Lipids**+**⁎FBG**+**RBG**	99.31±2.406; 99.28±2.510	96.01±6.011; 91.70±7.675	91.94±5.791; 92.60±3.813	96.08±2.007; 95.59±2.278
**Lipids**+**⁎FBG**+**HbA**_**1c**_	99.64±0.115; 99.70±0.135	98.22±0.240; 98.32±0.204	97.89±0.334; 97.99±0.336	97.76±0.305; 97.65±0.288
**Lipids**+**⁎FBG**+**RBG**+**HbA**_**1c**_	96.76±4.148; 97.69±3.199	93.71±6.317; 95.42±4.577	95.78±2.578; 95.33±3.774	96.71±1.188; 96.24±1.526

**GROUP**	**2014**	**2015**	**2016**
**Lipids**	90.79±1.221; 92.05±1.376	68.91±9.862; 75.11±8.236	51.91±2.584; 57.79±1.834
**Lipids**+**RBG**	65.06±4.100; 66.74±4.787	37.27±8.831; 41.56±6.119	28.16±1.465; 33.30±0.893
**Lipids**+**HbA**_**1c**_	84.33±2.626; 86.92±1.365	54.96±10.892; 63.28±9.707	39.11±1.315; 47.46±1.747
**Lipids**+**RBG**+**HbA**_**1c**_	76.29±3.385; 77.88±4.205	46.02±10.493; 50.90±8.681	30.90±1.076; 39.33±1.900
**Lipids**+**⁎FBG**	97.18±0.300; 96.60±0.271	95.69±0.420; 95.59±0.533	95.56±0.365; 95.18±0.417
**Lipids**+**⁎FBG**+**RBG**	93.89±1.989; 94.30±2.560	91.29±4.134; 92.58±3.398	93.55±3.555; 92.93±2.770
**Lipids**+**⁎FBG**+**HbA**_**1c**_	97.39±0.161; 97.40±0.218	96.24±0.273; 96.10±0.311	95.98±0.336; 95.79±0.285
**Lipids**+**⁎FBG**+**RBG**+**HbA**_**1c**_	95.07±1.416; 95.04±1.245	93.46±1.677; 92.43±2.144	91.38±0.942; 93.36±1.900

*Lipids=total cholesterol, high density lipoprotein, low density lipoprotein, triglycerides 2016 only has data from January 1–June 30; the rest are January 1–December 31*.

*⁎ - fasting required for test.*

*RBG=random blood glucose.*

*HbA*_*1c*_*=hemoglobin A*_*1c*_.

*FBG=fasting blood glucose*.

## 2 Experimental design, materials, and methods

The experimental design and methods have been described elsewhere [1]. Briefly, CLS is the sole supplier of laboratory services for Calgary and surrounding area in Alberta, Canada, performing on average 29 million tests per year for a population of approximately 1.4 million individuals. Nearly 99% of cholesterol tests processed by CLS are performed on community-based individuals, with the remaining 1% being performed on hospital-based patients.

The study population consisted of 3,003,667 community-based testing encounters (1,447,720 testing encounters for men and 1,555,947 testing encounters for women) ≥18 years of age, who received various lipid profile and diabetes screening-related tests between January 1, 2010 and June 30, 2016. The different types of tests are listed in Table 1, Table 2A, Table 2B, Table 3A and include: lipids, lipids+random blood glucose (RBG), lipids+hemoglobin A_1c_ (HbA_1c_), lipids+RBG+HbA_1c_, lipids+fasting blood glucose (FBG), lipids+FBG+RBG, lipids+FBG+HbA_1c_, and lipids+FBG+RBG+HbA_1c_. Time since last meal (hours fasted) was collected from all patients, where fasting was defined as ≥8 h since last meal [2], [3]. Counts of women and men were categorized based on fasting for tests that do not require fasting (Table 2A – women, Table 2B – men), and tests that require fasting (Table 3A – women, Table 3B – men). Data for individuals with undefined age at collection, sex, or fasting time was excluded in the data set.

**Table 2A:** Number of testing encounters for women fasting for various lipids and diabetes screening tests (fasting not required) in Calgary over time.

**Year**	**Month**	**Lipids**		**Lipids**+**RBG**		**Lipids**+**HbA1c**		**Lipids**+**RBG**+**HbA1c**	
		**Adults fasting (n)**	**Total adults (N)**	**Adults fasting (n)**	**Total adults (N)**	**Adults fasting (n)**	**Total adults (N)**	**Adults fasting (n)**	**Total adults (N)**
2010	January	1571	1587	305	317	337	340	3998	4024
2010	February	1415	1425	288	299	329	332	4206	4224
2010	March	1746	1757	347	362	403	413	5095	5113
2010	April	1523	1538	284	290	363	368	4593	4606
2010	May	1511	1522	288	299	367	371	4933	4944
2010	June	1673	1685	311	321	440	445	5099	5114
2010	July	1534	1539	279	285	391	395	4777	4787
2010	August	1511	1512	223	233	445	447	4476	4494
2010	September	1586	1591	239	250	473	481	4937	4957
2010	October	1583	1589	238	247	468	473	5308	5327
2010	November	1558	1564	258	271	388	390	4907	4922
2010	December	1362	1378	193	203	416	421	4237	4253
2011	January	1482	1541	218	248	398	412	4841	4945
2011	February	1384	1441	253	285	423	441	4434	4525
2011	March	1630	1707	325	374	525	557	5843	5957
2011	April	1457	1500	252	287	436	460	5623	5727
2011	May	1458	1509	217	237	439	459	6025	6153
2011	June	1625	1690	223	247	472	501	6098	6202
2011	July	1341	1382	197	226	429	448	5247	5344
2011	August	1395	1434	209	235	466	488	5305	5402
2011	September	1537	1572	224	249	526	549	5828	5933
2011	October	1533	1583	223	253	548	566	6000	6091
2011	November	1462	1500	221	254	528	557	6020	6103
2011	December	1289	1315	213	239	452	468	5027	5102
2012	January	1466	1507	193	217	535	559	5693	5792
2012	February	1522	1578	216	244	535	562	6015	6135
2012	March	1656	1728	341	383	648	688	6913	7056
2012	April	1605	1684	302	342	667	712	6134	6237
2012	May	1754	1845	392	440	779	844	6756	6878
2012	June	1783	1895	412	467	814	882	6727	6863
2012	July	1494	1573	344	402	773	833	5497	5614
2012	August	1715	1802	412	469	852	915	6244	6384
2012	September	1648	1724	377	436	819	886	6110	6275
2012	October	1709	1808	402	484	899	968	6713	6867
2012	November	1687	1776	357	435	858	931	6300	6475
2012	December	1417	1495	284	331	754	813	5204	5328
2013	January	1625	1718	394	461	1011	1132	6244	6416
2013	February	1492	1600	526	603	1008	1104	6534	6692
2013	March	1528	1640	485	562	1002	1126	6941	7122
2013	April	1692	1823	395	484	1221	1353	7758	7946
2013	May	1748	1868	352	462	1263	1433	8271	8450
2013	June	1524	1641	339	482	1183	1318	7739	7866
2013	July	1627	1729	310	460	1235	1381	7520	7659
2013	August	1522	1634	308	421	1134	1269	7788	7945
2013	September	1635	1761	314	429	1227	1383	7713	7897
2013	October	1693	1801	410	554	1369	1558	8507	8715
2013	November	1609	1749	346	462	1318	1540	7977	8171
2013	December	1286	1393	253	357	1099	1258	6276	6424
2014	January	1527	1686	312	459	1457	1717	8176	8419
2014	February	1411	1557	300	428	1447	1649	8159	8363
2014	March	1533	1671	292	413	1573	1828	8766	8994
2014	April	1590	1728	318	452	1775	2031	9325	9556
2014	May	1629	1785	297	451	1883	2216	9645	9901
2014	June	1640	1790	282	427	1898	2204	9389	9663
2014	July	1549	1692	240	378	1815	2168	9207	9461
2014	August	1592	1721	226	361	1874	2176	8610	8827
2014	September	1650	1839	272	426	2021	2432	9017	9267
2014	October	1806	2002	275	456	2447	3013	9699	9940
2014	November	1564	1769	253	419	2106	2600	8438	8675
2014	December	1512	1694	230	389	1881	2362	7826	8039
2015	January	1649	1870	221	396	2146	2728	8196	8493
2015	February	1449	1765	201	385	1943	2760	8078	8338
2015	March	1499	1962	201	504	2086	3345	9515	9867
2015	April	1422	1937	191	473	1951	3367	8945	9310
2015	May	1318	1851	208	538	1962	3509	8852	9192
2015	June	1356	1986	237	678	2010	3907	8861	9229
2015	July	1291	1937	217	674	1777	3442	8114	8416
2015	August	1122	1751	190	611	1695	3381	7218	7515
2015	September	1188	1883	225	689	1857	3878	7664	7983
2015	October	1191	1979	248	803	1887	4129	8211	8544
2015	November	984	1784	265	835	1650	3909	6955	7243
2015	December	1005	1727	195	729	1634	3614	6216	6471
2016	January	994	1797	261	923	1706	4114	6303	6608
2016	February	971	1811	267	919	1627	4221	6633	6916
2016	March	975	1909	293	1,015	1855	4780	7203	7511
2016	April	1036	1954	262	1,038	1930	4904	7094	7370
2016	May	925	1839	307	1,080	1983	5088	7068	7343
2016	June	960	1995	300	1,032	1992	5312	6900	7173
**Total**		**114,941**	**132,304**	**22,078**	**35,279**	**89,333**	**133,014**	**530,714**	**544,083**

**Table 2B:** Number of testing encounters for men fasting for various lipids and diabetes screening tests (fasting not required) in Calgary over time.

**Year**	**Month**	**Lipids**		**Lipids**+**RBG**		**Lipids**+**HbA1c**		**Lipids**+**RBG**+**HbA1c**	
		**Adults fasting (n)**	**Total adults (N)**	**Adults fasting (n)**	**Total adults (N)**	**Adults fasting (n)**	**Total adults (N)**	**Adults fasting (n)**	**Total adults (N)**
2010	January	1935	1947	337	349	411	413	4663	4689
2010	February	1709	1718	304	315	419	424	4588	4607
2010	March	1983	1994	318	330	495	498	5474	5490
2010	April	1842	1853	326	336	489	494	5142	5159
2010	May	1783	1790	290	297	481	483	5262	5270
2010	June	1921	1927	294	306	501	504	5296	5319
2010	July	1802	1809	245	254	459	462	4801	4812
2010	August	1720	1731	213	223	463	465	4741	4747
2010	September	1724	1730	243	249	506	511	5233	5246
2010	October	1919	1928	275	281	515	517	5464	5474
2010	November	1722	1727	280	286	576	583	5393	5404
2010	December	1717	1729	216	220	469	473	4926	4948
2011	January	1762	1813	230	262	523	546	5197	5300
2011	February	1534	1573	279	316	474	498	4753	4853
2011	March	1836	1890	344	391	614	634	5902	6008
2011	April	1695	1753	245	267	578	595	5808	5903
2011	May	1683	1719	226	251	534	551	6168	6285
2011	June	1780	1815	260	278	574	590	6107	6208
2011	July	1483	1531	229	254	446	466	5111	5178
2011	August	1650	1689	208	227	533	550	5247	5334
2011	September	1691	1725	241	266	516	538	5895	5987
2011	October	1627	1660	264	297	571	595	6158	6261
2011	November	1700	1755	232	261	656	677	6379	6481
2011	December	1717	1753	216	247	553	573	5832	5925
2012	January	1769	1809	252	277	654	679	6340	6440
2012	February	1662	1701	272	315	632	663	6337	6434
2012	March	1829	1884	345	389	744	775	6962	7088
2012	April	1836	1884	347	380	754	796	6533	6661
2012	May	1887	1981	417	478	904	958	6745	6884
2012	June	2002	2092	346	395	840	892	6731	6871
2012	July	1656	1717	295	351	816	865	5327	5430
2012	August	1756	1823	359	411	831	874	5892	6020
2012	September	1816	1894	418	467	866	916	5843	5990
2012	October	1964	2058	346	397	1023	1086	6631	6799
2012	November	1934	2024	342	388	969	1030	6468	6635
2012	December	1694	1778	271	310	901	975	5803	5913
2013	January	1791	1885	452	517	1048	1137	6589	6784
2013	February	1689	1788	732	808	989	1067	6285	6436
2013	March	1727	1834	406	472	1072	1159	7053	7233
2013	April	1959	2090	355	419	1197	1311	7730	7943
2013	May	1824	1956	314	407	1181	1288	7872	8046
2013	June	1706	1812	291	400	1055	1162	7457	7612
2013	July	1620	1728	275	370	1126	1244	7049	7178
2013	August	1575	1671	263	363	1121	1228	7051	7220
2013	September	1684	1793	292	386	1157	1321	7251	7424
2013	October	1829	1974	303	427	1369	1532	8166	8348
2013	November	1703	1833	289	408	1316	1470	8065	8276
2013	December	1622	1721	245	344	1222	1349	7008	7180
2014	January	1663	1820	283	397	1424	1622	8533	8783
2014	February	1528	1625	257	363	1422	1598	8114	8293
2014	March	1755	1874	270	381	1586	1802	8711	8927
2014	April	1754	1899	269	396	1689	1922	9296	9550
2014	May	1738	1871	235	345	1860	2126	9163	9430
2014	June	1737	1875	222	321	1737	1986	8918	9139
2014	July	1757	1896	218	302	1729	1974	8359	8569
2014	August	1579	1707	186	286	1671	1925	7870	8092
2014	September	1797	1969	200	315	1956	2269	8524	8742
2014	October	1894	2088	253	387	2332	2741	9154	9399
2014	November	1661	1867	204	357	2176	2554	8324	8552
2014	December	1825	1993	212	356	2158	2551	8323	8571
2015	January	1905	2097	186	362	2260	2731	8371	8654
2015	February	1546	1841	176	350	2044	2649	7988	8319
2015	March	1748	2120	175	376	2242	3127	9354	9741
2015	April	1641	2030	192	479	2046	3107	8842	9222
2015	May	1458	1918	180	385	2023	3133	8370	8679
2015	June	1563	2088	181	447	2051	3373	8592	8949
2015	July	1478	1961	206	505	1832	3032	7503	7797
2015	August	1290	1805	197	479	1794	2976	6749	7025
2015	September	1265	1830	200	505	1987	3515	7491	7809
2015	October	1310	1953	227	652	2065	3754	7723	8006
2015	November	1213	1866	217	667	1950	3746	6959	7264
2015	December	1166	1813	224	653	2041	3908	6946	7263
2016	January	1225	2055	268	807	1995	3958	6640	6931
2016	February	1174	1952	279	827	1858	3876	6581	6907
2016	March	1238	2187	267	831	2091	4549	7187	7489
2016	April	1264	2174	268	828	2106	4635	7040	7335
2016	May	1234	2172	285	834	2199	4640	6773	7081
2016	June	1267	2285	293	857	2346	4926	6562	6833
**Total**		**130,142**	**145,920**	**21,372**	**31,692**	**94,813**	**129,122**	**531,688**	**545,084**

**Table 3A:** Number of testing encounters for women fasting for various lipids and diabetes screening tests (fasting required) in Calgary over time.

**Year**	**Month**	**Lipids**+**FBG**		**Lipids**+**FBG**+**RBG**		**Lipids**+**FBG**+**HbA1c**		**Lipids**+**FBG**+**RBG**+**HbA1c**	
		**Adults fasting (n)**	**Total adults (N)**	**Adults fasting (n)**	**Total adults (N)**	**Adults fasting (n)**	**Total adults (N)**	**Adults fasting (n)**	**Total adults (N)**
2010	January	10,010	10,037	11	11	250	269	23	24
2010	February	9709	9734	5	5	249	262	10	10
2010	March	12,002	12,037	11	11	244	260	19	19
2010	April	10,761	10,788	11	11	228	237	15	15
2010	May	11,150	11,172	10	10	294	303	14	16
2010	June	11,220	11,238	14	14	210	218	17	17
2010	July	10,409	10,423	5	5	189	200	27	28
2010	August	9866	9890	11	12	142	145	20	22
2010	September	10,853	10,876	9	9	181	196	18	19
2010	October	11,188	11,214	20	20	179	188	22	23
2010	November	10,546	10,563	22	22	259	269	14	14
2010	December	8801	8820	16	16	204	218	16	16
2011	January	9517	9741	13	15	190	217	16	19
2011	February	9300	9511	7	7	281	311	22	23
2011	March	11,505	11,787	17	17	320	367	11	13
2011	April	10,753	10,968	8	9	274	303	14	14
2011	May	11,590	11,789	16	16	278	303	20	20
2011	June	11,613	11,788	10	11	275	290	7	8
2011	July	9714	9875	13	13	259	282	11	11
2011	August	10,186	10,341	8	8	241	258	11	11
2011	September	10,341	10,523	9	9	289	308	9	10
2011	October	10,558	10,723	5	5	206	231	11	12
2011	November	10,354	10,520	8	8	280	307	10	11
2011	December	8483	8621	6	7	240	263	9	9
2012	January	9407	9561	7	9	210	237	10	11
2012	February	9831	9999	12	13	309	337	16	17
2012	March	11,158	11,354	19	20	340	369	17	18
2012	April	9689	9880	30	32	517	551	33	34
2012	May	10,495	10,687	34	35	679	737	62	67
2012	June	9859	10,066	53	56	980	1052	106	106
2012	July	8055	8223	34	40	877	954	110	112
2012	August	8692	8883	68	72	1144	1221	94	97
2012	September	8293	8489	43	49	1260	1369	103	107
2012	October	9276	9502	77	84	1575	1692	118	124
2012	November	8734	8939	62	64	1664	1757	143	150
2012	December	6595	6776	63	65	1288	1378	123	125
2013	January	7616	7810	79	84	1308	1429	157	161
2013	February	7546	7766	78	83	1337	1492	148	156
2013	March	7746	7936	58	59	1598	1756	164	172
2013	April	8392	8606	83	89	905	1038	161	168
2013	May	8701	8872	104	105	939	1132	165	170
2013	June	7402	7520	75	79	843	1035	166	169
2013	July	7301	7425	81	83	889	1070	147	149
2013	August	6878	7016	70	73	841	1020	169	174
2013	September	6743	6900	85	87	818	1011	144	150
2013	October	7071	7245	93	98	900	1096	154	161
2013	November	6436	6577	57	60	828	1022	170	176
2013	December	4936	5074	60	61	699	845	124	127
2014	January	5974	6147	84	85	761	1007	165	176
2014	February	5968	6101	72	79	818	1029	198	206
2014	March	6378	6541	69	73	867	1093	205	209
2014	April	6753	6924	61	65	925	1184	185	197
2014	May	6800	6997	69	74	997	1284	199	211
2014	June	6944	7149	75	80	899	1148	193	202
2014	July	6227	6428	57	60	887	1139	206	212
2014	August	5825	5998	61	65	945	1175	191	202
2014	September	5730	5904	72	79	951	1287	197	208
2014	October	6133	6320	56	60	1024	1462	204	217
2014	November	5309	5484	56	59	949	1292	172	181
2014	December	4669	4818	54	58	892	1250	162	173
2015	January	4812	4989	60	67	959	1392	185	192
2015	February	4697	4895	48	53	918	1477	193	204
2015	March	5369	5610	55	57	903	1771	197	214
2015	April	4934	5150	58	63	863	1835	230	243
2015	May	4737	4927	39	39	987	2109	200	219
2015	June	4614	4812	68	74	1173	2612	214	229
2015	July	4075	4258	56	62	1110	2569	206	223
2015	August	3662	3838	52	61	1030	2463	204	222
2015	September	3605	3769	44	49	1167	2928	196	206
2015	October	3727	3903	53	56	1246	3339	218	239
2015	November	3365	3547	40	46	1113	3184	181	191
2015	December	2858	3002	44	50	1015	2963	179	191
2016	January	3082	3225	59	63	1063	3288	189	205
2016	February	3115	3279	70	71	1131	3518	168	184
2016	March	3263	3425	62	67	1121	3709	199	215
2016	April	3285	3427	44	49	1195	3920	166	183
2016	May	3061	3198	59	61	1189	3905	173	189
2016	June	2916	3039	63	70	1150	3867	162	180
**Total**		**589,168**	**601,189**	**3450**	**3666**	**58,758**	**97,004**	**8907**	**9408**

**Table 3B:** Number of testing encounters for men fasting for various lipids and diabetes screening tests (fasting required) in Calgary over time.

**Year**	**Month**	**Lipids**+**FBG**		**Lipids**+**FBG**+**RBG**		**Lipids**+**FBG**+**HbA1c**		**Lipids**+**FBG**+**RBG**+**HbA1c**	
		**Adults fasting (n)**	**Total adults (N)**	**Adults fasting (n)**	**Total adults (N)**	**Adults fasting (n)**	**Total adults (N)**	**Adults fasting (n)**	**Total adults (N)**
2010	January	8443	8463	12	12	316	329	28	28
2010	February	8233	8254	14	14	338	352	23	23
2010	March	9822	9853	4	4	377	436	32	34
2010	April	8854	8869	13	13	383	418	28	28
2010	May	8520	8528	15	15	383	406	29	29
2010	June	8716	8732	14	14	219	236	48	49
2010	July	7968	7979	9	9	179	186	32	35
2010	August	7478	7491	9	9	178	185	27	27
2010	September	8213	8224	10	10	175	179	20	21
2010	October	8831	8841	21	23	220	227	29	31
2010	November	8626	8642	18	18	346	381	16	16
2010	December	7705	7715	10	10	263	276	23	23
2011	January	8133	8344	12	14	259	285	25	27
2011	February	7713	7921	8	9	358	405	18	20
2011	March	9375	9581	9	9	401	440	25	26
2011	April	8768	8991	7	8	368	416	22	22
2011	May	8696	8884	7	8	260	278	15	17
2011	June	8848	9011	7	7	332	349	25	25
2011	July	7536	7672	12	12	393	418	18	20
2011	August	7785	7909	8	8	222	236	21	21
2011	September	7963	8119	8	10	355	388	13	14
2011	October	8393	8539	10	10	300	325	20	21
2011	November	8641	8783	7	8	329	352	13	13
2011	December	7690	7836	5	6	352	393	16	16
2012	January	7980	8126	5	5	237	255	16	16
2012	February	8025	8202	7	8	305	332	11	13
2012	March	9025	9198	17	18	380	405	16	17
2012	April	7678	7827	25	28	529	584	39	40
2012	May	8072	8250	41	45	710	782	54	55
2012	June	7385	7577	46	50	936	1015	87	90
2012	July	5989	6120	32	34	823	899	83	86
2012	August	6593	6762	57	63	977	1054	86	90
2012	September	6406	6585	50	55	1109	1204	98	104
2012	October	7272	7455	39	44	1366	1491	124	130
2012	November	7317	7517	62	63	1451	1560	129	136
2012	December	6097	6262	50	53	1254	1342	133	138
2013	January	6545	6683	69	74	1321	1443	169	174
2013	February	6247	6432	70	72	1345	1483	144	149
2013	March	6442	6644	47	49	1413	1542	155	163
2013	April	6936	7128	79	84	923	1040	194	204
2013	May	6711	6831	68	70	866	1049	186	196
2013	June	5751	5860	87	90	761	926	139	142
2013	July	5359	5451	64	68	782	940	153	156
2013	August	5343	5448	77	85	753	925	145	147
2013	September	5173	5304	63	64	751	926	151	156
2013	October	5542	5679	76	78	846	1026	173	180
2013	November	5280	5420	91	96	799	983	183	195
2013	December	4745	4874	77	79	719	867	161	170
2014	January	5315	5487	88	92	832	1068	201	215
2014	February	4959	5102	95	98	797	962	177	190
2014	March	5308	5477	87	94	848	1029	220	228
2014	April	5546	5686	71	79	849	1077	203	215
2014	May	5426	5590	73	78	914	1195	221	233
2014	June	5473	5651	68	72	850	1047	194	202
2014	July	5031	5192	65	69	811	1028	192	198
2014	August	4607	4767	69	70	743	911	192	199
2014	September	4811	4979	58	63	906	1163	219	230
2014	October	5137	5312	63	66	977	1291	222	236
2014	November	4728	4895	63	65	848	1233	195	205
2014	December	4581	4725	74	81	895	1241	193	205
2015	January	4517	4677	60	67	936	1335	237	249
2015	February	4154	4321	49	54	830	1316	208	222
2015	March	4572	4776	56	61	953	1646	233	255
2015	April	4250	4448	44	48	924	1804	219	231
2015	May	4078	4268	43	48	942	1850	245	262
2015	June	3914	4078	55	62	1027	2179	232	250
2015	July	3465	3611	50	52	1091	2197	255	269
2015	August	2957	3091	60	62	972	2052	201	220
2015	September	3086	3248	40	40	1087	2399	205	226
2015	October	3368	3538	56	60	1186	2715	206	224
2015	November	3086	3257	62	68	1143	2698	165	188
2015	December	2952	3107	54	59	1132	2692	216	237
2016	January	2962	3112	79	82	1291	3047	181	200
2016	February	2921	3090	79	84	1211	3105	194	211
2016	March	3092	3252	78	84	1320	3326	214	224
2016	April	3110	3245	74	84	1365	3423	217	229
2016	May	2918	3064	70	76	1219	3173	173	186
2016	June	2635	2768	80	85	1176	3213	168	178
**Total**		**481,822**	**492,630**	**3511**	**3738**	**58,737**	**89,384**	**9593**	**10,150**

For each month during the study period, the proportion of men and women fasting for the various lipid and diabetes screening-related tests was calculated by dividing the total number of individuals fasting (*n*) by the total number of individuals fasted that month (*N*). As documented in Table 1, the total mean±standard deviation (SD) was determined for each gender for the proportion of individuals fasting for each year (GraphPad Prism, version 6, San Diego, CA).
